# Expression and Prognostic Value of miR-486-5p in Patients with Gastric Adenocarcinoma

**DOI:** 10.1371/journal.pone.0119384

**Published:** 2015-03-20

**Authors:** Hui Chen, Chuanli Ren, Chongxu Han, Daxin Wang, Yong Chen, Deyuan Fu

**Affiliations:** 1 Geriatric Medicine, Northern Jiangsu People’s Hospital and Clinical Medical College of Yangzhou University, Yangzhou, China; 2 Clinical Medical Testing Laboratory, Northern Jiangsu People’s Hospital and Clinical Medical College of Yangzhou University, Yangzhou, China; 3 Departments of Oncology, Northern Jiangsu People’s Hospital and Clinical Medical College of Yangzhou University, Yangzhou, China; 4 Breast Oncology Surgery, Northern Jiangsu People’s Hospital and Clinical Medical College of Yangzhou University, Yangzhou, China; 5 Department of Epidemiology and Biostatistics, Ministry of Education (MOE) Key Laboratory of Modern Toxicology, School of Public Health, Nanjing Medical University, Nanjing, China; Sapporo Medical University, JAPAN

## Abstract

MicroRNA (miR)-486-5p expression is often reduced in human cancers. However, its expression in gastric carcinoma and its relation to clinicopathological features and prognosis are unclear. Tissue microarrays were constructed from 84 patients with gastric adenocarcinoma (GC) who were undergoing radical resection. miR-486-5p expression was detected by miRNA-locked nucleic acid *in situ* hybridization, and its correlations with clinicopathological features and overall survival were analyzed. Bioinformatic studies predict that fibroblast growth factor 9 (FGF9) is a potential target gene of miR-486-5p. miR-486-5p was mainly located in the cytoplasm of GC cells and neighboring normal tissues. Compared with paracancerous normal tissue, miR-486-5p expression was decreased in 63.1% (53/84) of the GC samples, increased in 32.1% (27/84) and unchanged in 4.8% (4/84). FGF9 expression was decreased in 69.0% (58/84) of GC samples and increased in 31.0% (26/84) compared with normal paracancerous tissues using immunohistochemical analysis. Low or unchanged miR-486-5p expression (*P* = 0.002), tumor stage (*P* = 0.001), tumor status (*P* = 0.001), node status (*P* = 0.001), tumor size (*P* = 0.004), and depth of tumor invasion (*P* = 0.013) were significant negative prognostic predictors for overall survival in patients with GC. After stratification according to American Joint Committee on Cancer (AJCC) stage, low/unchanged miR-486-5p expression remained a significant predictor of poor survival in stage II (*P* = 0.024) and stage III (*P* = 0.003). Cox regression analysis identified the following predictors of poor prognosis: tumor status (hazard ratio [HR], 7.19; 95% confidence interval [CI], 1.75–29.6; P = 0.006), stage (HR, 2.62; 95%CI, 1.50–4.59; P = 0.001), lymph node metastasis (HR, 2.52; 95% CI, 1.27–4.99; *P* = 0.008), low/unchanged miR-486-5p (HR, 2.47; 95% CI, 1.35–4.52; *P* = 0.003), high level of FGF9 (HR, 2.41; 95% CI, 1.42–4.09; *P* = 0.001) and tumor size (HR, 2.50; 95% CI, 1.30–4.82; *P* = 0.006). Low or unchanged expression of miR-486-5p compared with neighboring normal tissues was associated with a poor prognosis, while high expression was associated with a good prognosis in GC. miR-486-5p may thus be useful for evaluating prognosis and may provide a novel target treatment in patients with GC.

## Introduction

Gastric cancer (GC) is currently the fourth most common cancer and the second highest cause of cancer-related mortality worldwide, accounting for an estimated 989,000 new cases and 738,000 deaths in 2008 [1]. However, nearly half the global GA incidence (464,000) and deaths (352,000) occur in China [1]. It is generally accepted that gastric carcinogenesis, like that of other malignancies, involves the accumulation of genetic and epigenetic changes [2]. However, the precise mechanisms underlying gastric carcinogenesis and prognosis remain unclear.

MicroRNAs (miRNAs) act as post-transcriptional regulators that can repress translation or promote degradation or cleavage of complementary target mRNA sequences [3]. A single miRNA can influence the expression of hundreds of target genes, and miRNAs have been reported as key regulatory molecules in various diseases, including cancer [4]. Numerous studies have demonstrated that various miRNAs function as potential oncogenes or tumor suppressor genes during the progression of cancer [5].

Reduced miR-486–5p expression is a frequent molecular event in human cancers [6–13]. Deregulation of cancer-related miR-486–5p can be a common event in both benign and malignant human breast tumors[13]. However, miR-486–5p was upregulated in renal cell carcinoma tissue [14]. We previously systematically screened the plasma miRNA profiles of patients with gastric non-cardia adenocarcinoma and healthy controls and identified miR-486–5p as one of five differentially expressed miRNAs [15]. miR-486–5p has been reported to be a potentially useful biomarker for GC [16], but there is an apparent contradiction between miR-486–5p expression in plasma and in GC tissues [15]. miR-486–5p is one of the most downregulated miRNAs in lung tumor tissues and contributes to lung cancer progression and metastasis through regulating Rho GTPase-activating protein 5 (ARHGAP5)[17]. Serum miR-486–5p has great potential for further applications in the clinical diagnosis of lung cancers[18]. miR-486 was significantly downregulated in primary GC and GC cell lines, and genomic loss of the miR-486 locus has been shown in approximately 25–30% of GC, which is consistent with a tumor-suppressive role [11]. However, the clinical significance of miR-486–5p in GC remains unknown.

The fibroblast growth factor (FGF) family consists of at least 23 polypeptides that have important functions in embryonic development, tissue repair, and tumorigenesis [19]. Using bioinformatics analysis, we hypothesize that FGF9 may be one of the target genes of miR-486–5p. We therefore investigated expression of miR-486–5p in GA by miRNA-locked nucleic acid (LNA) *in situ* hybridization and FGF9 protein expression by immunohistochemistry and evaluated its relationship with pathological parameters and prognosis.

## Materials and Methods

### Patients and tissue samples

Paraffin-embedded tissue samples were collected retrospectively from archival material stored in the Biobank Center at the National Engineering Center for Biochip at Shanghai. Samples from tumor tissue and corresponding neighboring normal tissue were collected from 90 patients with histologically diagnosed GC who underwent surgical resection between 2004 and 2008.

The following clinicopathological data were obtained from the original pathology reports: age, sex, tumor size, location and invasion, lymph node metastases, grade of differentiation, and tumor stage. Staging of GC was assessed according to the AJCC criteria. The clinical and pathological data for the patients is provided in Table 1. Written informed consent was obtained from all patients, and the protocol was approved by the Ethical Committee of the National Engineering Center for Biochip at Shanghai.

**Table 1:** Characteristics of the study subjects.

Clinicopathologic features	Number	Percentage (%)
**Age (years)**		
**<60**	27	32.1
**≥60**	57	67.9
**Gender**		
**male**	65	77.4
**female**	19	22.6
**Tumor Size (cm)**		
**<10**	13	15.5
**≥10**	71	74.5
**Tumor site**		
**cardia**	16	19.0
**body**	14	16.7
**antrum**	36	42.9
**others**	18	21.4
**Pathological type**		
**adenocarcinoma**	84	100
**Nodal status**		
**negative**	22	26.2
**positive**	62	73.8
**Tumor stage**		
**I**	7	8.3
**II**	29	34.5
**III**	44	52.4
**IV**	4	4.8
**Follow-up time (months)**	38 (1–75)	
**Prognosis**		
**alive**	25	29.8
**dead**	59	70.2
**patients lived for 5 years**	28	33.3

Follow-up times were measured from the date of surgery to the date of death for all 90 GC patients. Data for six patients were excluded because the dots were off the chips in this experiment. Data for a total of 84 patients with GC were therefore included in the final analysis. The last follow-up point was in March 2013, at which point 84 patients had been followed up and were included in the 5-year survival analysis. The median follow-up time was 38 months (range 1–75 months). Among the 84 patients, 46 died during the follow-up period.

### Tissue microarray construction

Tissue microarrays (TMAs) were constructed using appropriate tissue cores from formalin-fixed and paraffin-embedded samples as described previously [20]. Briefly, the appropriate tumor areas and corresponding non-tumor gastric samples were selected by pathologists, and a single core (diameter 0.6 mm) was taken from each tissue. TMA blocks were constructed using an automated tissue arrayer (Beecher Instruments, Sun Prairie, WI, USA). The array blocks were cut into 5-μm sections, and the sections were stained with hematoxylin and eosin to verify the presence of tumor cells. In all cases, tissue cores obtained from normal adjacent tissue served as internal controls.

### miRNA-LNA *in situ* hybridization

miRNA-LNA *in situ* hybridization was performed using antisense oligonucleotide probes for miR-486–5p (Exiqon Inc., Woburn, MA, USA) with Scramble-miR as a negative control. The sections were deparaffinized, hydrated and deproteinated, followed by prehybridization in hybridization buffer for 2 h in a humidified chamber at 55°C. Hybridization was performed by applying 20 nM of probe in hybridization buffer to the array slides covered with Nescofilm (Bando Chemical Co., Kombe, Japan) overnight at 55°C in a humidified chamber. Hybridized probes were detected by incubation with anti-digoxigenin and alkaline phosphatase conjugate at 37°C for 30 min, followed by nitro blue tetrazolium/5-bromo-4-chloro-3-indolyl-phosphate to develop a blue color. Finally, the cells were counterstained with nuclear fast red for 3–5 min and mounted on slides. Sections with ≤5% labeled cells were scored as 0; sections with 5–30% labeled cells were scored as 1; sections with 31–70% labeled cells were scored as 2; and sections with ≥71% of labeled cells were scored as 3. The staining intensity was scored similarly, with 0 indicating negative staining, 1 indicating weakly positive, 2 indicating moderately positive and 3 indicating strongly positive. The scores for the percentage of positive tumor cells and staining intensity were summed to generate an immunoreactive score for each specimen. A final score of 0–1 indicated negative expression (−), 2–3 indicated weak expression (+), 4–5 indicated moderate expression (++), and 6 indicated strong expression (+++). Each sample was examined separately and scored by two pathologists [21]. In our research, high levels of miR-486–5p in GC samples indicate that the final score of miR-486–5p expression in GC is higher than that of normal paracancerous tissues. Low levels of miR-486–5p in GC indicate that the final score of miR-486–5p expression in GC samples is higher than that of normal paracancerous tissues. Unchanged levels of miR-486–5p in GC samples indicate that the final score of miR-486–5p expression in GC samples is equal to that of normal paracancerous tissues.

### Immunohistochemistry

Immunohistochemical analysis was performed for 90 GC specimens. All tumor tissues and the surrounding gastric tissues were removed and embedded in paraffin and cut into 4-cm thick sections. These sections were deparaffinized, rehydrated, and incubated in 0.03% H_2_O_2_ in 95% methanol at room temperature for 20 min to block endogenous peroxidase activity. Antigen retrieval was performed using water bath pretreatment (Immunosaver; Nisshin EM, Tokyo, Japan) at 98°C for 45 min. All sections were incubated for 20 min with normal horse serum to eliminate non- specific staining and were then incubated with anti-human FGF9 antibody (#ab71395, Abcam Cambridge, UK) overnight at 4°C. This step was followed by incubation with the secondary antibody (Imm- PRESS Reagent Kit; Vector Laboratories, Burlingame, CA) for 30 min. Slides were then incubated in diaminobenzidine (DAB)/Trissolution (3DAB/Tris) tablets diluted in 150 ml of distilled water; Muto Pure Chemicals, Tokyo, Japan) supplemented with 15 μl of 30% H_2_O_2_. Finally, the slides were counterstained with hematoxylin. The proportion of cells stained and the staining intensity score were assessed by the pathologist as follows: 0, absence of staining; 1, weakly stained; 2, moderately stained; and 3, strongly stained. The total score was calculated by multiplying the proportion score with the intensity score[22].

### Bioinformatics

Two software programs, TargetScan 5.2 (Release 5.2, June 2011; www.targetscan.org/) and miRecords (http://mirecords.biolead.org/), were used to predict the potential target genes of miR-486–5p.

### Statistical analysis

Associations between clinicopathological parameters and miR-486–5p expression were evaluated using χ^2^ tests. When sample numbers in some categorical cells were less than 5, Fisher's exact test was employed. Overall survival was calculated and survival curves were plotted using the Kaplan—Meier method; differences between groups were compared using log-rank tests. Significant variables in univariate models were further analyzed by multivariate Cox proportional hazards regression models to identify the independent prognostic values. All analyses were performed using the SPSS software package (SPSS Inc, Chicago, IL, USA, version 17.0). All tests were two-sided and *P* values < 0.05 were considered statistically significant.

## Results

### Aberrant expression of miR-486–5p in GA and paracancerous tissues

**Fig 1:**
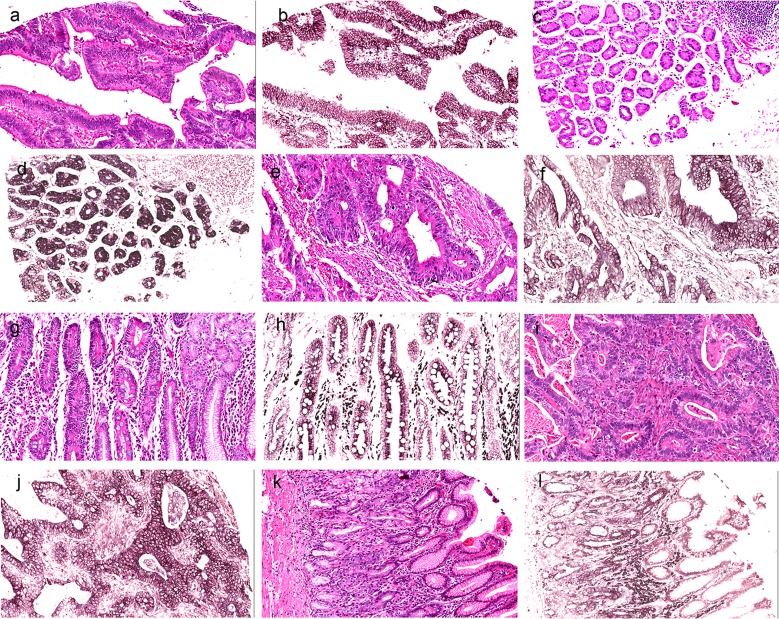
miRNA-486–5p levels were stained by *in situ* hybridization. **a** Hematoxylin and eosin (HE)-stained GC tissue. **b** miR-486–5p stained by *in situ* hybridization. **c** HE-stained neighboring normal tissue. **d** miR-486–5p stained by *in situ* hybridization; decreased miR-486–5p expression in GC compared with neighboring normal tissue. **e** HE-stained GC tissue. **f** miR-486–5p stained by *in situ* hybridization. **g** HE-stained neighboring normal tissue. **h** miR-486–5p stained by *in situ* hybridization; unchanged miR-486–5p expression in GC compared with neighboring normal tissue. **i** HE-stained GC. **j** miR-486–5p stained by *in situ* hybridization. **k** HE-stained neighboring normal tissue. **l** miR-486–5p stained by *in situ* hybridization; increased miR-486–5p expression in GC compared with neighboring normal tissue.

*In situ* hybridization showed that miR-486–5p was mainly located in the cytoplasm of the GA cells and the normal paracancerous tissues (Fig. 1). Compared with the normal paracancerous tissues, miR-486–5p expression was decreased in 63.1% (53/84), increased in 32.1% (27/84), and unchanged in 4.8% (4/84) of the GA samples. Aberrant miR-486–5p expression was thus detected in most GA tissues, and miR-486–5p was decreased in most cases of GA (*P* < 0.01).

### Relationships between miR-486–5p expression and clinicopathological features in GA

There was a tendency towards a difference in TNM stage between patients with low/unchanged levels of miR-486–5p expression and patients with high expression (χ^2^ = 2.912, *P* = 0.088), but no significant correlations were found between miR-486–5p expression levels and other clinicopathological variables, including age, sex, tumor site, TNM stage, tumor size, nodal status, distant metastasis and depth of tumor invasion (Table 2).

**Table 2:** miR-486-5p expression and clinicopathological features in patients with gastric adenocarcinoma.

Characteristics	miR-486–5p low or unchanged expression n (%)	miR-486–5p high expression n (%)	χ2 or Fisher's exact test	P-value
**Age (years)**			4.673	0.031
**<60**	14(24.6)	13(48.1)		
**≥60**	43(75.4)	14(51.9)		
**Gender**			1.117	0.291
**male**	46(80.7)	19(70.4)		
**female**	11(19.3)	8(29.6)		
**Local invasion**			2.912	0.088
**T1+T2**	5 (8.8)	6 (22.2)		
**T3+T4**	52 (91.2)	21 (77.8)		
**Site**			1.625	0.202
**gastric cardia**	13 (22.8)	3(11.1)		
**non-cardia**	44 (77.2)	24(88.9)		
**TNM stage**			0.455	0.5
**I + II**	23 (40.3)	13 (48.1)		
**III + IV**	34 (59.7)	14 (51.9)		
**Nodal status**			2.422	0.120
**positive**	45(78.9)	17(63.0)		
**negative**	12(21.1)	10(37.0)		
**Distant metastasis**				0.591
**M0**	55(96.5)	25(92.6)		
**M1**	2(3.5)	2(7.4)		
**Tumor size (cm)**				1.0
**≥10**	9(15.8)	4(14.8)		
**<10**	48(84.2)	23(85.2)		
**Depth of tumor invasion**			0.136	0.712
**Mucosa, submucosa, muscularis propria, subserosa**	40(70.2)	20(74.1)		
**Penetration of serosa, adjacent structures**	17(29.8)	7(25.9)		

### Fibroblast growth factor (FGF) 9 was a potential target of miR-486–5p

Two software programs, TargetScan 5.2 (Release 5.2, June 2011; www.targetscan.org/) and miRecords (http://mirecords.biolead.org/), were used to predict the potential target genes of miR-486–5p. FGF9 was selected as a potential target gene of miR-486–5p in patients with GA.

### Expression of FGF9 protein in patients with GA

**Fig 2:**
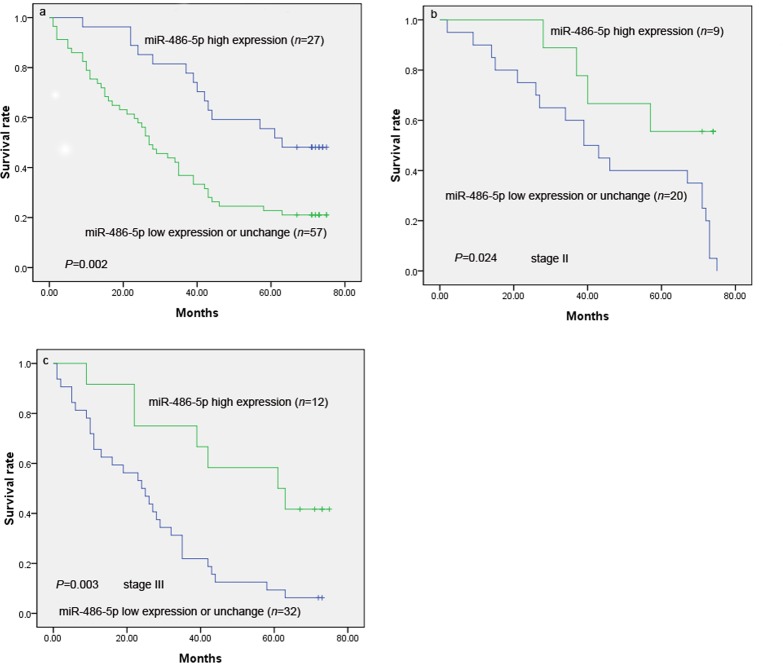
FGF9 expression was stained by immunohistochemistry and survival curves in patients with GC according to FGF9 levels. **a** High expression of FGF9 stained in GC. **b** Low expression FGF9 stained in neighboring normal tissue. **c** Survival curves in patients with high expression of FGF9 in GC compared with patients with low expression of FGF9 (*P* = 0.001).

Immunohistochemistry staining showed that FGF9 was mainly located in the cytoplasm of the cells and the normal paracancerous tissues (Fig. 2). In the samples, FGF9 expression was decreased in 69.0% (58/84) and increased in 31.0% (26/84) compared with the normal paracancerous tissues.

### Survival analysis

The median overall survival (OS) in the study cohort was 38 months, and the longest was 75 months. Kaplan—Meier analysis demonstrated that low or unchanged expression of miR-486–5p, stage of disease, tumor status, node status, tumor size, and depth of tumor invasion were significant negative prognostic predictors for OS in patients with (*P* = 0.002, *P* < 0.001, *P* = 0.001, *P* < 0.001, *P* = 0.004, and *P* = 0.013, respectively). Other clinicopathological characteristics, including age, sex, and tumor size and location, were not significantly associated with prognosis. Only four patients with distant metastases were included in the study; therefore, the prognostic significance of distant metastasis could not be calculated (*P* = 0.12, Table 3).

**Table 3:** Univariate analysis of survival.

Variable	Mean survival time Month (±SE)	95% CI(Month)	*P*
**Age (years)**			0.806
**<60**	39.6(5.3)	29.3–50.0	
**≥60**	41.6(3.4)	35.0–48.2	
**Gender**			
**Male**	41.0(3.3)	34.5–47.4	0.962
**Female**	40.7(5.7)	30.0–51.8	
**Tumor site**			0.362
**Gastric cardia**	36.4 (5.3)	26.0–46.9	
**Non-cardia**	42.0 (3.3)	35.6–48.4	
**Stage of disease**			<0.001
**I–II**	52.0(4.2)	43.7–60.3	
**III–IV**	32.7 (3.4)	26.1–39.4	
**Tumor status (p)**			0.001
**T1-T2**	65.5 (6.7)	52.4–78.5	
**T3-T4**	37.3 (2.9)	31.6–43.0	
**Node status**			<0.001
**Negative**	55.8 (2.9)	27.4–40.5	
**Positive**	34.0 (3.3)	50.0–61.5	
**Distant metastasis**			0.120
**No**	25.0 (6.6)	12.0–38.0	
**Yes**	41.8(3.0	36.0–47.6	
**MiR-486–5p**			0.002
**High expression**	55.8 (4.1)	47.7–63.9	
**Low/unchanged**	34.0 (3.3)	27.4–40.5	
**Tumor size (cm)**			0.004
**≥10**	25.1 (4.8)	15.6–34.5	
**<10**	43.9 (3.1)	35.4–46.6	
**Depth of tumor invasion**			0.013
**Mucosa, submucosa, muscularis propria, subserosa**	30.3(4.5)	21.5–39.1	
**Penetration of serosa, adjacent structures**	45.1(3.4)	38.5–51.8	

**Fig 3:**
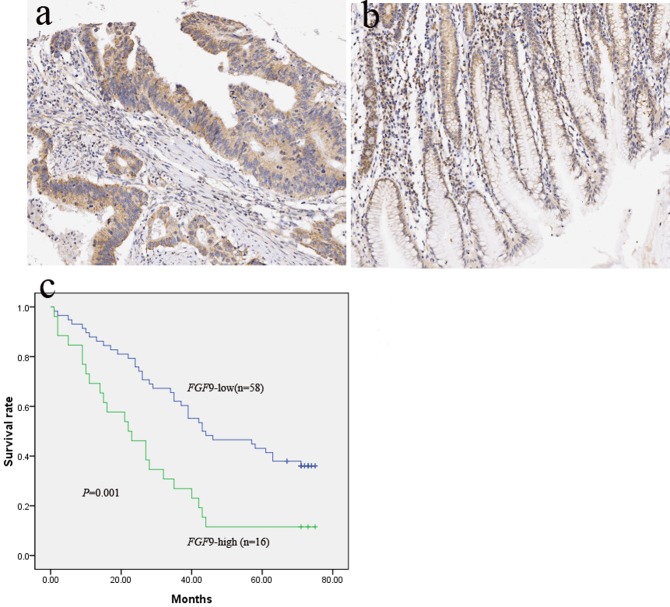
Survival curves in patients with GC according to miRNA-486–5p levels. **a** Overall survival curves in patients with GC according to miRNA-486–5p levels (*P* = 0.002). **b** Survival curves in stage-II GC according to miRNA-486–5p levels (*P* = 0.024). **c** Survival curves in stage-III GC according to miRNA-486–5p levels (*P* = 0.003).

The prognosis of patients with low/unchanged miR-486–5p expression was significantly worse than that of patients with high miR-486–5p expression (*P* = 0.002; Fig. 3). The median survival times were 55.8 months for high miR-486–5p expression and 34.0 months for low/unchanged miR-486–5p expression. After stratification of patients according to AJCC stage, low/unchanged miR-486–5p expression remained a significant predictor of poor survival in stage-II (*P* = 0.024, *n* = 29) and stage-III (*P* = 0.003, *n* = 44). Variables that were significantly associated with OS in univariate analysis were included in the Cox proportional hazards multivariate regression analysis. Tumor status (hazard ratio [HR], 7.19; 95% confidence interval [CI], 1.75–29.6; *P* = 0.006), stage (HR, 2.62; 95% CI, 1.50–4.59; *P* = 0.001), lymph node metastasis (HR, 2.52; 95% CI, 1.27–4.99; *P* = 0.008), low/unchanged miR-486–5p expression (HR, 2.47; 95% CI, 1.35–4.52; *P* = 0.003) and tumor size (HR, 2.50; 95% CI, 1.30–4.82; *P* = 0.006) predicted poor prognosis. Age (HR, 0.897; 95% CI, 0.52–1.56; *P* = 0.701), sex (HR, 1.02; 95% CI, 0.55–1.88; *P* = 0.962) and tumor site (HR, 1.33; 95% CI, 0.72–2.46; *P* = 0.368) were not related to prognosis (*P* = 0.368). Cox regression analysis demonstrated that high level of FGF9 (HR, 2.41; 95% CI, 1.42–4.09; *P* = 0.001) predicted poor overall survival (Table 4).

**Table 4:** Multivariate Cox regression analysis of potential prognostic factors for survival in patients with GC.

Variables	Univariate analysis		Multivariate analysis	
	HR(95%CI)	P-value	HR(95%CI)	P-value
**Tumor status, T3-T4 vs. T1-T2**	7.19(1.75–29.6)	0.006		0.038
**Stage, III-IVvs. I–II**	2.62(1.50–4.59)	0.001		0.015
**LNM, yes vs. no**	2.52(1.27–4.99)	0.008	2.37(1.16–4.86)	0.019
**Low/unchanged miR-486–5p vs. high miR-486–5p**	2.47(1.35–4.52)	0.003	3.61(1.99–6.54)	0.001
**High FGF9 vs. Low FGF9**	2.41(1.42–4.09)	0.001	2.31(1.32–4.05)	0.003
**Tumor size (cm), ≥10 vs. <10**	2.50(1.30–4.82)	0.006		0.005
**Age (years),<60 vs. ≥60**	0.897(0.52–1.56)	0.701		0.067
**Gender,male vs. female**	1.02(0.55–1.88)	0.962		0.770
**Tumor site,**gastric cardia vs**. non-cardia**	1.33(0.72–2.46)	0.368		0.822

## Discussion

miRNAs comprise a class of non-coding RNAs that function as negative gene regulators. Through binding with partially complementary sequences in the 3′-untranslated regions of targeted mRNAs, individual miRNAs can simultaneously regulate a variety of target genes involved in the development and progression of human cancers and other non-malignant diseases. To date, the anti-apoptotic OLFM4 [11], SIRT1 [23], PIM-1[24], DOCK3[25]and the tumor suppressor PTEN [26] have been reported as potential targets for miR-486. However, the mechanistic role of miR-486 as either an oncogene or a tumor suppressor in remains largely unknown.

Hsa-miR-486 (miR-486) was significantly downregulated in primary and cell lines, and restoration of miR-486 expression in cell lines caused suppression of several pro-oncogenic traits, whereas its inhibition enhanced cellular proliferation [11], suggesting a tumor-suppressive role for miR-486. miR-486 has also been found to be upregulated in invasive pancreatic cancer cell lines [27]. This upregulation may be specific to the pancreatic cell lines used or may be cancer-specific, owing to the cell-specific availability of mRNA targets such as miR-31[28]. In addition to the increase in circulating miR-486–5p in the plasma of GC and lung cancer samples in our previous study[15,29], saliva and saliva supernatant miR-486–5p were significantly upregulated and showed great promise as biomarkers for the detection of esophageal cancer[30]. However, the expression and prognostic value of miR-486–5p in patients with resected GC is unknown.

We compared miR-486–5p expression levels in GC samples and paracancerous tissues by miRNA-LNA *in situ* hybridization and showed that miR-486–5p expression was significantly decreased in GC overall. However, its expression was increased in 32.1% of samples and unchanged in 4.8%, suggesting that the prognostic implication of miR-486–5p expression may vary. In recent years, activation of FGF/FGFR signals through FGF9 has been reported in some cancers. Leushacke et al. reported that the expression level of FGF9 mRNA was high in a subset of resected non-small cell lung cancer and that FGF9 high expression was negatively correlated with patient survival[31]. Deng et al. found that miR-26a suppresses tumor growth and metastasis by targeting FGF9 in gastric cancer[32].

FGF 9 is a potential target gene of miR-486–5p predicted by bioinformatics in our research. Low miR-486–5p expression in GC has a significant relationship with high expression of FGF 9 protein (data not shown). We found that high level of FGF9 in patients with GA predicted poor overall survival (Fig. 2). Moreover, univariate and multivariate analyses showed that high expression of FGF 9 is an independent predictor of overall survival (HR, 2.41; 95% CI, 1.42–4.09; *P* = 0.001; HR, 2.31; 95% CI, 1.32–4.05; *P* = 0.003).

To the best of our knowledge, this study is the first to systematically explore the role of miR-486–5p in the clinicopathological features and prognosis of GC, with a goal of identifying curative therapies. Navon and colleagues recently reported that miR-486 was underexpressed in several other types of cancer [8]. However, we found overexpression of miR-486–5p in 32.1% of GC patients. A previous four-phase study detected increased miR-486–5p expression during the early stage of GC and identified five plasma miRNAs (miR-16, miR-25, miR-92a, miR-451 and miR-486–5p) that might serve as potential diagnostic markers for early-stage gastric non-cardia adenocarcinoma [15] However, Goto and colleagues found that miR-486–5p was unregulated in renal cell carcinoma, and high miR-486 expression in tumors was associated with worse cancer-specific mortality[14]. Our research group previously identified miR-486–5p as one of the four serum miRNA signatures that may serve as a noninvasive predictor of overall survival in NSCLC[29]. In this study, GC patients with higher miR-486–5p expression showed better prognoses than those patients with lower or unchanged expression. Moreover, multivariate Cox regression analysis of potential prognostic factors for GC survival identified a significant relationship between low/unchanged miR-486–5p expression and poor prognosis (*P* = 0.003). However, the results for miR-486–5p expression in plasma and GC tissues seem contradictory. This apparent discrepancy between miR-486–5p levels in tissues and the circulation may be explained by selective release of specific cellular miRNAs from tumor cells or normal cells. However, the detection of miR-486–5p in the circulation or tissues may provide new information to help in the early diagnosis and prognostic evaluation of GC, though the molecular function of miR-486–5p in GC is unknown.

miR-486–5p expression levels were significantly lower in lung tumors compared with their corresponding normal tissues[17]. miR-486–5p may act as a tumor suppressor, contributing to the progression and metastasis of non-small cell lung cancer by targeting ARHGAP5[17]. A previous study demonstrated that analyzing miR-486–5p in sputum and plasma specimens could provide a diagnostic approach for the early detection of lung cancer [33]. miR-486–5p was found to be downregulated in breast cancer patients with lymph node metastases[34] and to exert its antiproliferative function and promote apoptosis by directly downregulating PIM-1 expression [24]. There are several possible explanations for the observed downregulation of miR-486–5p in tumor tissues. For example, miR-486–5p is located on chromosome 8p11.21, which is one of the most frequently deleted genomic regions containing potential tumor suppressor genes in various types of tumors, including GC and lung cancer [11,17]. miR-486 can directly target components of insulin-like growth factor (IGF) signaling, including IGF1, and functions as a potent tumor suppressor of lung cancer both *in vitro* and *in vivo* [12]. Several components of IGF signaling, including IGF1R, p85α, and IGF1, are direct targets of miR-486, which causes reductions in downstream pAKT and pFoxo3a [12]. miR-486–5p is also associated with bromocriptine-resistant prolactinoma [35].

The results of this study indicate that gastric adenocarcinomas with low or unchanged levels of miR-486–5p expression compared with neighboring normal tissues have a relatively poor prognosis, while high miR-486–5p expression is associated with a better prognosis. Cox regression analysis identified low levels of miR-486–5p and high expression of FGF9 to be independent risk factors for overall survival in GC. miR-486–5p may possess the potency to suppress FGF9 protein expression. Although further studies are needed to confirm these findings, the results suggest that miR-486–5p may represent a valuable prognostic indicator as well as a potential target for the treatment of GC.
